# Editorial: Bacterial Chromosomes Under Changing Environmental Conditions

**DOI:** 10.3389/fmicb.2021.633466

**Published:** 2021-03-11

**Authors:** Monika Glinkowska, Torsten Waldminghaus, Leise Riber

**Affiliations:** ^1^Department of Bacterial Molecular Genetics, University of Gdansk, Gdańsk, Poland; ^2^Department of Biology, Technische Universität Darmstadt, Darmstadt, Germany; ^3^Centre for Synthetic Biology, Technische Universität Darmstadt, Darmstadt, Germany; ^4^Department of Plant and Environmental Sciences, University of Copenhagen, Copenhagen, Denmark

**Keywords:** bacterial chromosomes, nucleoid architecture, DNA replication, chromosome segregation, DNA repair, stress conditions, environmental conditions, replication-transcription conflicts

The bacterial cell cycle comprises chromosome replication and segregation of newly replicated chromosomes into daughter cells prior to cell division. Unlike in eukaryotic organisms, DNA replication, chromosome segregation, and transcription occur simultaneously in bacteria. Several molecular mechanisms act in concert to allow chromosome replication initiation once-and-only-once per cell cycle (Skarstad et al., 1986; Boye et al., 2000). Other mechanisms ensure that replication is coordinated with cell growth (Murray, 2016) and linked to chromosome segregation in a tightly coordinated manner (Blow and Tanaka, 2005; Reyes-Lamothe et al., 2012). Considering that the chromosome is a massively compact structure, organization of the bacterial nucleoid adds an extra level to cell cycle coordination. In particular, a balance has to be reached between the requirement of significant compaction and an unobstructed accessibility to molecular processes underlying essential cellular functions, such as replication, transcription, DNA repair and homologous recombination (Badrinarayanan et al., 2015; Magnan and Bates, 2015).

As single cell organisms, bacteria constantly adapt to ever changing environmental conditions. Typical examples of adverse environmental conditions include exposure to antibiotics, nutrient limitation, changes in physical parameters (pH, temperature, osmotic pressure), exposure to ultraviolet (UV) radiation or oxidative stress. As such conditions might influence control mechanisms governing chromosome dynamics (Golovlev, 2003; Tymeca-Mulik et al., 2017; Remesh et al., 2020), bacterial cells have developed highly advanced survival strategies to overcome the negative impact of the encountered stress. Those strategies enable them to adapt to unfavorable conditions, thereby allowing colonization of various ecological niches including host organisms (Boor, 2006; Hauryliuk et al., 2015). Previously, the acquisition of foreign DNA was suggested to impact on nucleoid structure as well (Krogh et al., 2018). However, the molecular mechanisms of how chromosome maintenance systems cope with such challenges and potentially help to overcome significant threats to genome stability, cell cycle regulation and viability, remain poorly understood.

This Research Topic represents a comprehensive collection of articles focusing on the influence of changing environmental conditions on bacterial chromosome dynamics, such as chromosome organization, replication, segregation and DNA repair.

On the topological level, dynamic organization of the nucleoid involves a tight balance between efficient compaction within the cell and concomitant accessibility to replication, transcription and DNA repair processes. Original research by Krogh et al. demonstrates the importance of chromosome architecture on transcriptional regulation in bacteria. Co-expression of genes was found to correlate positively with increased spatial proximity. This suggests that nucleoid structure, through the ability to bring genes together, strongly influences the amount of transcriptional spilling into neighboring genes.

Changing environmental conditions are known to induce profound topological alterations of the chromosome structure. NAPs (nucleoid associated proteins) have been shown to play a major role in adjusting bacterial chromosome architecture and affecting gene transcription regulation in response to stress, as reviewed by Holowka and Zakrzewska-Czerwinska.

Besides nucleoid organization, replication, transcription and DNA repair processes are by themselves sensitive to changing environmental conditions. In this context, Sinha et al. review the current understanding of molecular mechanisms underlying the negative impact of the stringent stress response regulator, (p)ppGpp, on chromosome replication initiation in *Escherichia coli*, and on chromosome replication elongation in *Bacillus subtilis*, respectively. In addition, they propose a model where (p)ppGpp-mediated survival during DNA damage is linked to the ability of (p)ppGpp to inhibit replication initiation, which minimizes the frequency of replication-transcription collisions, hence allowing backtracking of RNA polymerase to repair genotoxic DNA lesions. An additional facet of stringent response-mediated regulation of DNA replication initiation in bacteria is the polyphosphate-induced proteolysis and degradation of essential replication proteins, as reviewed by Ropelewska et al.

In most bacteria, DnaA-*oriC* dependent replication initiation is considered an essential mechanism. However, Ohbayashi et al. demonstrate variations in cyanobacterial chromosome replication mechanisms, manifested by regular/irregular GC skew profiles. Here, the genomes of certain free-living species are found not to encode *dnaA*, and instead chromosome replication in those organisms is initiated from multiple origins in a DnaA-independent manner. This replication mode produces irregular GC skew profiles, indicating that loss of DnaA-*oriC* dependencies might play a crucial role in cyanobacterial evolution.

In the context of elucidating novel DNA replication processes, Oliveira Paiva et al. revealed that a pathogen *Clostridioides difficile* utilizes a bipartite origin of replication, possibly conserved among *Clostridioides* species. Within this origin DnaA-dependent unwinding occurs at *oriC2*, in the *dnaA-dnaN* intergenic region.

During normal growth conditions, chromosome replication progression is tightly coordinated with simultaneous gene transcription. If, however, additional origins are engineered into different ectopic genomic locations, native replichore arrangements are disturbed and genome trafficking events, such as replication-transcription conflicts, might arise. Syeda et al. review current models of how such replication-transcription conflicts contribute to shaping of the distinct architecture of bacterial chromosomes.

Replication-transcription collisions also induce multiple repair pathways required to restart arrested replication forks. A novel insight into DNA repair mechanisms is brought by Sheng et al. who report that two *recA* variants are induced by UV in *Myxococcus xanthus* cells, each playing a different role in cell growth and UV-radiation resistance. Most bacteria, including *E. coli*, possess a single *recA* gene, and duplicate *recA* genes have been investigated only in *Bacillus megaterium* and *Myxococcus xanthus*. The findings of this research article, therefore, add a valuable insight onto the functional divergence among duplicated *recA* genes in bacteria.

Finally, chromosome segregation constitutes an essential stage of cell cycle progression, and ParA and ParB are known as main players in cellular positioning of the replication origin prior to cell division in most bacterial species. However, ParA and ParB have been shown to interact with proteins involved in cell division or cell elongation. Based on this, Pioro and Jakimowicz review evidence on the regulatory role of segregation proteins in cell cycle progression and cover the current understanding of its coordination with environmental conditions.

In conclusion, this Research Topic highlights a selection of original research- and review articles representing the current progress within understanding molecular survival strategies adopted by bacteria to preserve cell cycle regulation and genome integrity in response to changing environmental conditions. We kindly thank all contributors, authors as well as reviewers, for their valuable time, thoughts and input for this Research Topic published in the section of Evolutionary and Genomic Microbiology in Frontiers in Microbiology. We hope that the readers will enjoy their work as much as we have.

## Author Contributions

MG, TW, and LR were joint co-editors of this Research Topic and co-wrote the editorial. All authors contributed equally to this article and approved the submitted version.

## Conflict of Interest

The authors declare that the research was conducted in the absence of any commercial or financial relationships that could be construed as a potential conflict of interest.
